# Clinical factors associated with outcome in solid tumor patients treated with immune-checkpoint inhibitors: a single institution retrospective analysis

**DOI:** 10.1007/s12672-022-00538-6

**Published:** 2022-08-12

**Authors:** Qian Qin, Tomi Jun, Bo Wang, Vaibhav G. Patel, George Mellgard, Xiaobo Zhong, Mahalya Gogerly-Moragoda, Anish B. Parikh, Amanda Leiter, Emily J. Gallagher, Parissa Alerasool, Philip Garcia, Himanshu Joshi, MBBS, Matthew Galsky, William K. Oh, Che-Kai Tsao

**Affiliations:** 1 Division of Hematology/Medical Oncology, Tisch Cancer Institute, Icahn School of Medicine at Mount Sinai, 1470 Madison Avenue, Third Floor Admin Suite #108 (Hess Building), New York, NY USA; 2 Icahn School of Medicine at Mount Sinai, New York, NY USA; 3 Department of Population Health and Policy, Icahn School of Medicine at Mount Sinai, New York, NY USA; 4 Division of Medical Oncology, The Ohio State University Comprehensive Cancer Center-James Cancer Hospital, Columbus, OH USA; 5 Division of Endocrinology, Diabetes and Bone Disease, Department of Medicine, Icahn School of Medicine at Mount Sinai, New York, NY USA; 6 New York Medical College, Valhalla, NY USA; 7 Institute for Healthcare Delivery Science, Icahn School of Medicine at Mount Sinai, New York, NY USA

**Keywords:** Immune checkpoint inhibitors, Solid tumors, Clinical prognosticators of response, Performance status, Bone metastasis

## Abstract

**Objectives:**

Response to immune checkpoint inhibitor (ICI) remains limited to a subset of patients and predictive biomarkers of response remains an unmet need, limiting our ability to provide precision medicine. Using real-world data, we aimed to identify potential clinical prognosticators of ICI response in solid tumor patients.

**Methods:**

We conducted a retrospective analysis of all solid tumor patients treated with ICIs at the Mount Sinai Hospital between January 2011 and April 2017. Predictors assessed included demographics, performance status, co-morbidities, family history of cancer, smoking status, cancer type, metastatic pattern, and type of ICI. Outcomes evaluated include progression free survival (PFS), overall survival (OS), overall response rate (ORR) and disease control rate (DCR). Univariable and multivariable Cox proportional hazard models were constructed to test the association of predictors with outcomes.

**Results:**

We identified 297 ICI-treated patients with diagnosis of non-small cell lung cancer (N = 81, 27.3%), melanoma (N = 73, 24.6%), hepatocellular carcinoma (N = 51, 17.2%), urothelial carcinoma (N = 51, 17.2%), head and neck squamous cell carcinoma (N = 23, 7.7%), and renal cell carcinoma (N = 18, 6.1%). In multivariable analysis, good performance status of ECOG ≤ 2 (PFS, ORR, DCR and OS) and family history of cancer (ORR and DCR) associated with improved ICI response. Bone metastasis was associated with worse outcomes (PFS, ORR, and DCR).

**Conclusions:**

Mechanisms underlying the clinical predictors of response observed in this real-world analysis, such as genetic variants and bone metastasis-tumor microenvironment, warrant further exploration in larger studies incorporating translational endpoints. Consistently positive clinical correlates may help inform patient stratification when considering ICI therapy.

**Supplementary Information:**

The online version contains supplementary material available at 10.1007/s12672-022-00538-6.

## Introduction

The introduction of immune checkpoint inhibitors (ICIs) has drastically changed the landscape of cancer therapeutics. First approved in the treatment of advanced melanoma, programmed cell death protein 1 (PD-1), programmed death-ligand 1 (PD-L1), and cytotoxic T-lymphocyte-associated protein 4 (CTLA-4) inhibitors either alone or in combination are now widely utilized in a variety of tumors [1–3].

However, given the dynamic and complex nature of immune responses, identifying predictors of response to ICIs remains challenging. The clinical utility of biomarkers such as PD-L1 expression, tumor mutation burden (TMB), tumor infiltrating lymphocytes, and liquid biopsy biomarkers such as peripheral blood neutrophil-to-lymphocyte ratio or circulating tumor DNA, varies greatly among cancer types and treatment settings [3, 4]. Clinical characteristics such as demographics, performance status, comorbidities, and metastatic sites may play crucial roles, but are less well-defined in published literature [3, 5]. For example, prior studies have evaluated the effect of obesity on response to ICI, although data remains inconclusive [3]. Similarly, other clinical factors such as age, gender, race, smoking history, and performance status have also been evaluated in both the pre-clinical and clinical settings, but their effects on ICI response also remains inconclusive [6–12].

In this study, we aim to identify potential, clinical characteristics that may be associate with efficacy outcomes in solid tumor patients receiving ICIs, with the goal of informing larger retrospective analyses to then instruct future clinical trial stratifications. Specifically, we retrospectively analyzed the correlations among host, cancer and treatment characteristics with ICI response in advanced, solid tumor patients treated with PD-1, PD-L1, and/or CTLA-4 inhibitors. Utilizing a heterogenous group of real-world patients, we aim to identify common characteristics that may be associated with ICI response regardless of underlying tumor type and/or other co-factors.

## Materials and methods

### Study design and setting

This is a retrospective cohort study involving adult cancer patients treated with ICIs at The Mount Sinai Hospital’s Tisch Cancer Institute between January 1, 2011 and April 28, 2017. Patients were followed until the censoring date of December 2018.

The primary outcome was progression-free survival (PFS), defined as the time from ICI initiation to radiographic progression, clinical progression, death from any cause, or loss to follow-up. Radiographic progression was defined as progressive disease per Response Evaluation Criteria in Solid Tumors version 1.1 (RECIST v1.1) criteria based on review of the radiology reports [13]. Clinical progression was assessed from manual review of clinic notes. Secondary outcomes were overall survival (OS), overall response rate (ORR), and disease control rate (DCR). ORR was defined as the proportion of patients attaining a best radiographic response of complete response (CR) or partial response (PR), DCR was defined as the proportion of patients attaining a CR, PR or stable disease (SD), per RECIST v1.1 criteria, and OS was defined as the time from ICI initiation to death from any cause [13]. Death was ascertained per chart review and/or review of web-based death registries.

Predictors assessed included age, sex, race/ethnicity, body mass index, Eastern Cooperative Oncology Group (ECOG) performance status, estimated glomerular filtration rate, family history of cancer, smoking status, cancer type, sites and number of metastases, line of treatment, and class of ICI agent (anti-PD1/PDL1, anti-CTLA4, or ICI-ICI combinations).

The Mount Sinai Hospital is tertiary referral academic care center in New York City. This study was approved by the Institutional Review Board of the Icahn School of Medicine at Mount Sinai.

### Patient cohort and data collection

Using a query of the cancer center immunotherapy database, we retrospectively identified all adult cancer patients who received ICI treatment [PD-1, PD-L1, and/or CTLA-4 inhibitor(s)] in the locally advanced, unresectable or metastatic setting at the Mount Sinai Hospital between January 2011 and April 2017. Patients who received only one dose of ICI were excluded as these were associated with being lost to follow-up, enrolment in hospice care, and other factors resulting in insufficient clinical data for analysis. Clinical data and outcomes were extracted from the electronic medical record via chart review of oncology clinical notes as well as laboratory, pathology, and radiology reports.

Study data were collected and managed using Research Electronic Data Capture (REDCap) tools hosted at the Icahn school of Medicine at Mount Sinai. REDCap is a secure, web-based software platform designed to support data capture for research studies [14, 15].

### Statistical analysis

Baseline characteristics were summarized using descriptive statistics. Continuous variables were summarized using medians and ranges, while categorical variables were summarized using counts and proportions. Patients were stratified by primary cancer type and each group was compared against all other patients to identify differences in baseline characteristics. Continuous variables were compared using a two-sided t-test or the Wilcoxon rank-sum test, while categorical variables were compared using the chi-squared test or Fisher’s exact test, as appropriate.

Median survival was estimated using the Kaplan–Meier method and compared using the log-rank test. Univariable and multivariable Cox proportional hazard models were constructed to test the association of predictors with PFS and OS. Predictors for the final multivariable models were selected in the following manner: we first conducted an univariate analysis using each candidate predictor. Then, because there were significant differences in baseline demographics between cancer types and because prognosis differs significantly by cancer type, we conducted a multivariate analysis adjusting for age, sex, and tumor type, plus each candidate predictor. Predictors which were significantly (p < 0.05) associated with survival in either the univariate analysis or the age/sex/tumor-adjusted analysis were then considered for the final model. The final selection of predictors was then based on the total number of events in the cohort, possible correlation of predictors, and clinical relevance. In all regression models, patients missing predictor or outcome data were excluded. We assessed the proportional hazards assumption for each model by testing for independence of the scaled Schoenfeld residuals and time. Age was included as a stratification variable in the final multivariable overall survival model due to violation of the proportional hazards assumption. Multicollinearity was assessed using variance inflation factors (VIF). The VIF value > 5 is an indicator of high multicollinearity. There was no evidence of multicollinearity in the multivariable models, VIF < 5. Hazard ratios (HR) and 95% confidence intervals (CI) were reported for the predictors in each model.

Regression analysis for the outcomes of overall response and disease control was conducted using logistic regression models. A similar approach to variable selection as described for the survival analyses was used for these outcomes. We reported the results of the regression models as odds ratios (OR) and their corresponding 95% CIs for each predictor in these models.

Subgroup analysis was performed to evaluate the association of bone metastases on PFS in different patient subgroups and to assess for effect modification within the subgroups. The analysis was performed by including an interaction term between bone metastases and each subgroup variable, in the Cox proportional hazards models. We estimated HRs and 95% CIs for bone metastases in each subgroup level from these models. Presence of effect modification was assessed based on the significance of the interaction term in each model.

All statistical analysis was done in R version 4.0.0, in conjunction with the *tidyverse, survival,* and *Publish* packages. Statistical significance was defined as a two-sided p-value < 0.05.

## Results

The cohort consisted of 297 ICI-treated patients, of which 7 (2%) had locally advanced, unresectable disease and 290 (98%) had metastatic disease. The types of ICI used include nivolumab (46.5%), ipilimumab (19.2%), pembrolizumab (17.5%), atezolizumab (14.1%), nivolumab/ipilimumab (2.4%), and durvalumab/tremelimumab (0.3%). The most common primary malignancy was non-small cell lung cancer (NSCLC; N = 81, 27.3%), followed by melanoma (N = 73, 24.6%), hepatocellular carcinoma (HCC; N = 51, 17.2%), urothelial carcinoma (UC; N = 51, 17.2%), head and neck squamous cell carcinoma (HNSC; N = 23, 7.7%), and renal cell carcinoma (RCC; N = 18, 6.1%). Overall, there were 139 deaths (46.8%) over a median follow-up of 350 days. Survival outcomes by primary malignancy are illustrated in Supplemental Table 1 and Supplemental Figure 1 with a median PFS of 128 days and median OS of 663 days. The pooled ORR was 27.6%; the pooled DCR was 41.8%.

### Baseline characteristics by primary malignancy

Patients’ baseline characteristics differed by primary malignancy (Table 1). UC patients were older than the rest of the cohort (median age 72, p = 0.001). There was a male preponderance in the cohort overall (63.6%); sex was more equally balanced among NSCLC patients than the rest of the cohort (male 49.4%, p = 0.003). A preserved performance status (ECOG 0–1) was less common in NSCLC than other cancers (77.3%, p = 0.001), but more common in melanoma than other cancers (95.4%, p = 0.05). Smoking history was more common in NSCLC than other cancers (84%, p < 0.001).

**Table 1:** Baseline characteristics by cancer histology

	NSCLC (N = 81)	Melanoma (N = 73)	Urothelial (N = 51)	HCC (N = 51)	Head & Neck (N = 23)	RCC (N = 18)	Overall (N = 297)
Age (yrs)	65 (48–83)	67 (21–95)	72 (33–91)*	66 (31–88)	68 (38–93)	63 (26–86)	67 (21–95)
Male	40 (49.4%)*	42 (57.5%)	38 (74.5%)	38 (74.5%)	19 (82.6%)	12 (66.7%)	189 (63.6%)
Non-Hispanic white	33 (40.7%)*	52 (71.2%)*	34 (66.7%)*	11 (21.6%)*	16 (69.6%)	12 (66.7%)	158 (53.2%)
Non-Hispanic black	18 (22.2%)*	3 (4.1%)*	3 (5.9%)	9 (17.6%)	3 (13%)	0 (0%)	36 (12.1%)
Hispanic	11 (13.6%)	7 (9.6%)	4 (7.8%)	5 (9.8%)	1 (4.3%)	4 (22.2%)	32 (10.8%)
Other race/ethnicity	19 (23.5%)	11 (15.1%)*	10 (19.6%)	26 (51%)*	3 (13%)	2 (11.1%)	71 (23.9%)
ECOG 0–1	58 (77.3%)*	62 (95.4%)*	45 (90%)	45 (91.8%)	22 (100%)	12 (80%)	244 (88.4%)
Body mass index (kg/m^2^)	22.9 (14.7–50)	25.8 (17.2–42)*	25 (19.5–36)	24.8 (16.2–40)	22.1 (15.4–37)*	28.3 (22.1–42)*	24.85 (14.7–50)
Family history of cancer	41 (50.6%)	42 (57.5%)*	17 (33.3%)	18 (35.3%)	10 (43.5%)	8 (44.4%)	136 (45.8%)
Current/former smoker	68 (84%)*	38 (52.1%)*	38 (74.5%)	36 (70.6%)	16 (69.6%)	6 (33.3%)*	202 (68%)
Reduced est. GFR†	17 (21.5%)	7 (9.6%)*	29 (56.9%)*	5 (9.8%)*	4 (17.4%)	12 (66.7%)*	74 (25.1%)
Metastatic sites							
Locally advanced	5 (6.2%)	3 (4.1%)	2 (3.9%)	12 (23.5%)*	0 (0%)	0 (0%)	22 (7.4%)
≥ 3 metastatic sites	26 (32.1%)	30 (41.1%)*	17 (33.3%)	2 (3.9%)*	5 (21.7%)	4 (22.2%)	84 (28.3%)
Lung metastases	–	39 (53.4%)	25 (49%)	18 (35.3%)*	14 (60.9%)	14 (77.8%)*	121 (49%)
Liver metastases	17 (21%)	20 (27.4%)	8 (15.7%)	–	4 (17.4%)	2 (11.1%)	58 (22.2%)
Lymph node metastases	55 (67.9%)	47 (64.4%)	34 (66.7%)	15 (30.6%)*	13 (56.5%)	12 (66.7%)	176 (59.7%)
Bone metastases	35 (43.2%)	21 (28.8%)	20 (39.2%)	13 (25.5%)	6 (26.1%)	7 (38.9%)	102 (34.3%)
CNS metastases	17 (21.2%)*	15 (20.5%)*	0 (0%)*	0 (0%)*	1 (4.3%)	1 (5.6%)	34 (11.5%)
Other metastases	17 (21%)	29 (39.7%)*	17 (37%)	6 (11.8%)*	4 (19%)	6 (40%)	79 (27.5%)
Treatment							
ICI given first line	19 (23.5%)*	62 (84.9%)*	23 (45.1%)	23 (45.1%)	8 (34.8%)	2 (11.1%)*	137 (46.1%)
ICI given on trial	2 (2.5%)*	6 (8.2%)*	34 (66.7%)*	3 (5.9%)*	6 (26.1%)	0 (0%)	51 (17.2%)
PD1/PDL1	81 (100%)*	18 (24.7%)*	42 (82.4%)	51 (100%)*	22 (95.7%)*	18 (100%)*	232 (78.1%)
CTLA-4	0 (0%)*	48 (65.8%)*	8 (15.7%)	0 (0%)*	1 (4.3%)	0 (0%)*	57 (19.2%)
ICI-ICI combination	0 (0%)	7 (9.6%)*	1 (2%)	0 (0%)	0 (0%)	0 (0%)	8 (2.7%)
ICI-chemo combination	0 (0%)*	0 (0%)	9 (17.6%)*	1 (2%)	1 (4.3%)	0 (0%)	11 (3.7%)

*CNS* central nervous system; *CTLA-4* cytotoxic T-lymphocyte-associated protein 4; *ECOG* Eastern Cooperative Oncology Group; *est* estimated; *GFR* glomerular filtration rate; *HCC* hepatocellular carcinoma; *HNSC* head and neck squamous cell carcinoma; *ICI* immune checkpoint inhibitor; *NSCLC* Non-small cell lung cancer; *PD-1* programmed cell death protein 1; *PD-L1* programmed death-ligand 1 *RCC* renal cell carcinoma; *UC* urothelial carcinoma, *yrs* years

*p < 0.05 compared to all others

†Reduced estimated glomerular filtration rate < 60 ml/min/1.73m^2^

Most patients were treated in the setting of metastatic disease, but treatment in the setting of locally advanced disease was more common in HCC than other cancers (23.5%, p < 0.001). Metastases involving the central nervous system were more commonly observed among NSCLC (21.2%, p = 0.003) and melanoma (20.5%, p = 0.01), as compared to metastasis among other cancers.

Across the different primary tumor types, ICI was administered as a first-line therapy most commonly in melanoma (84.9%, p < 0.001) and less commonly in RCC (11.1%, p = 0.002) and NSCLC (23.5%, p < 0.001) when compared with other cancer types. Melanoma patients were more likely to receive CTLA-4 inhibitors compared to other cancers (65.8%, p < 0.001). Similarly, combination immunotherapy regimens were used more commonly among melanoma patients as compared to the other cancers (9.59%, p < 0.001). ICI-chemotherapy combination was administered more commonly in UC patients as compared to other cancers (17.6%, P < 0.001).

### Factors associated with progression-free survival

In univariate Cox regression analyses, preserved performance status (HR 0.56, 95% CI 0.38–0.84), body mass index (HR 0.97, 95% CI 0.95–0.99), family history of cancer (HR 0.71, 95% CI 0.55–0.93), and presence of locally advanced disease (vs. metastatic; HR 0.54, 95% CI 0.29–0.98) were associated with improved PFS while presence of liver metastases (evaluated in non-HCC cancers only; HR 1.4, 95% CI 1–2), and bone metastases (HR 1.7, 95% CI 1.3–2.2) were associated with worse PFS. Because there were significant differences in baseline demographics between tumor types and because prognosis varies significantly by tumor type, we also performed a multivariable analysis adjusting for age, sex, and primary malignancy. All significant univariate predictors remained associated with PFS in the age/sex/tumor-adjusted analysis. Additionally, smoking history (HR 0.73, 95% CI 0.54–0.99) and reduced renal function (HR 0.7, 95% CI 0.49–0.99) were associated with PFS after adjusting for age, sex, and tumor.

In the final multivariable model incorporating all these predictors (except liver metastases), preserved performance status was associated with improved PFS (ECOG 0–1; HR 0.57, 95% CI 0.37–0.88) while bone metastases (HR 1.4, 95% CI 1–1.9) were associated with worse PFS (Table 2). When excluding HCC patients, liver metastases were not independently associated with PFS (HR 1.4, 95% CI 0.94–2.2).

**Table 2:** Cox Proportional Hazards Regression Models for Progression-Free Survival and Overall Survival

	Progression-Free Survival			Overall Survival		
Variable	Unadjusted HR (95% CI)	Age, sex, tumor- adjusted HR (95% CI)	Multivariable model HR† (95% CI)	Unadjusted HR (95% CI)	Age, sex, tumor- adjusted HR (95% CI)	Multivariable model HR† (95% CI)
Age > 70	0.91 (0.7–1.2)	0.85 (0.65–1.1)	0.91 (0.68–1.2)	0.93 (0.65–1.3)	0.93 (0.65–1.3)	–
Male	1.2 (0.91–1.6)	1.2 (0.89–1.6)	1.2 (0.91–1.7)	1.2 (0.84–1.7)	1.1 (0.79–1.7)	1.1 (0.75–1.7)
Non-Hispanic white	Ref	Ref	–	Ref	Ref	–
Non-Hispanic black	0.71 (0.46–1.1)	0.68 (0.43–1.1)	–	0.83 (0.48–1.4)	0.66 (0.37–1.2)	–
Hispanic	1.2 (0.81–1.9)	1.3 (0.8–2)	–	0.99 (0.57–1.7)	0.93 (0.52–1.6)	–
Other race/ethnicity	1.1 (0.8–1.5)	1 (0.71–1.4)	–	0.94 (0.61–1.4)	0.73 (0.46–1.2)	–
ECOG 0–1	0.56 (0.38–0.84)*	0.52 (0.34–0.8)*	0.57 (0.37–0.88)*	0.52 (0.32–0.84)*	0.45 (0.27–0.77)*	0.46 (0.27–0.8)*
Body mass index	0.97 (0.95–0.99)*	0.97 (0.95–1)*	0.98 (0.95–1)	0.98 (0.95–1)	0.99 (0.96–1)	–
Family history of cancer	0.71 (0.55–0.93)*	0.73 (0.56–0.96)*	0.84 (0.63–1.1)	0.71 (0.51–0.99)*	0.74 (0.53–1.1)	–
Current or former smoker	0.81 (0.61–1.1)	0.73 (0.54–0.99)*	0.77 (0.57–1.1)	1.3 (0.88–1.9)	1.1 (0.73–1.7)	–
Est. GFR < 60	0.84 (0.62–1.1)	0.7 (0.49–0.99)*	0.72 (0.5–1)	0.94 (0.64–1.4)	1 (0.67–1.6)	–
Locally advanced	0.53 (0.29–0.98)*	0.46 (0.24–0.88)*	0.52 (0.26–1)	0.43 (0.19–0.98)*	0.34 (0.15–0.78)*	0.4 (0.16–1)
≥ 3 metastatic sites	1.3 (0.99–1.7)	1.4 (1–1.9)*	1.2 (0.83–1.6)	1.5 (1.1–2.2)*	1.8 (1.2–2.6)*	1.6 (1–2.3)*
Lung metastases	1.1 (0.84–1.5)	1.1 (0.86–1.5)	–	1 (0.73–1.5)	1.2 (0.82–1.7)	–
Liver metastases	1.4 [1–2]*	1.5 (1.1–2.1)*	–	1.7 (1.1–2.6)*	1.6 (1.1–2.5)*	–
Lymph node metastases	1 (0.77–1.3)	1 (0.75–1.3)	–	1.1 (0.77–1.5)	1.2 (0.82–1.7)	–
Bone metastases	1.7 (1.3–2.2)*	1.7 (1.3–2.3)*	1.4 (1–1.9)*	1.7 (1.2–2.3)*	1.7 (1.2–2.5)*	1.3 (0.85–1.8)
CNS metastases	1.1 (0.74–1.6)	1.1 (0.74–1.8)	–	1.2 (0.7–1.9)	1.3 (0.76–2.2)	–
Other metastases	0.93 (0.69–1.3)	0.95 (0.7–1.3)	–	1.1 (0.78–1.7)	1.3 (0.87–1.9)	–
ICI given first line	0.95 (0.73–1.2)	1.1 (0.84–1.5)	–	0.94 (0.67–1.3)	1.1 (0.76–1.6)	–
ICI given on trial	1 (0.73–1.4)	0.88 (0.57–1.4)	–	0.93 (0.6–1.4)	0.87 (0.5–1.5)	–
PD1/PDL1	Ref	Ref	–	Ref	Ref	–
CTLA-4	1 (0.73–1.4)	1.6 (0.95–2.5)	–	0.76 (0.48–1.2)	1.1 (0.56–2.2)	–
ICI combination	0.74 (0.33–1.7)	1.2 (0.48–3.1)	–	0.61 (0.19–1.9)	0.89 (0.25–3.2)	–

*CI* confidence interval; *CNS* central nervous system; *CTLA-4* cytotoxic T-lymphocyte-associated protein 4; *ECOG* Eastern Cooperative Oncology Group; *est* estimated; *GFR* glomerular filtration rate; *ICI* immune checkpoint inhibitor; *HR* hazard ratio; *PD-1* programmed cell death protein 1; *PD-L1* programmed death-ligand 1, *Ref* reference

*p < 0.05

† Multivariable models also adjusted for tumor histology

### Factors associated with overall survival and response

In Cox regression analyses examining OS, better performance status and locally advanced disease (vs. metastatic) were associated with improved OS while 3 or more metastatic sites, liver metastases, and bone metastases were associated with worse OS in both univariate and age/sex/tumor-adjusted models (Table 2). In the final model adjusting for these predictors (except liver metastases), only preserved performance status (ECOG 0–1) was independently associated with OS (HR 0.46, 95% CI 0.27–0.8). When excluding HCC patients, liver metastases were not independently associated with OS (HR 1.3, 95% CI 0.74–2.2).

In addition, using logistic regression models to assess potential predictors of ORR and DCR, we identified preserved performance status (OR 7.39, 95% CI 1.6–35), family history of cancer (OR 1.93, 95% CI 1–3.6), and bone metastases (OR 0.41, 95% CI 0.21–0.81), as independently associated with overall response (Supplemental Table 2). Moreover, preserved performance status (OR 3.67, 95% CI 1.3–11), family history of cancer (OR 2.14, 95% CI 1.2–3.8), and bone metastases (OR 0.45, 95% CI 0.24–0.83) were also independently associated with disease control rate (Supplemental Table 3).

### Association of bone metastases with outcome across subgroups

**Figure 1.**
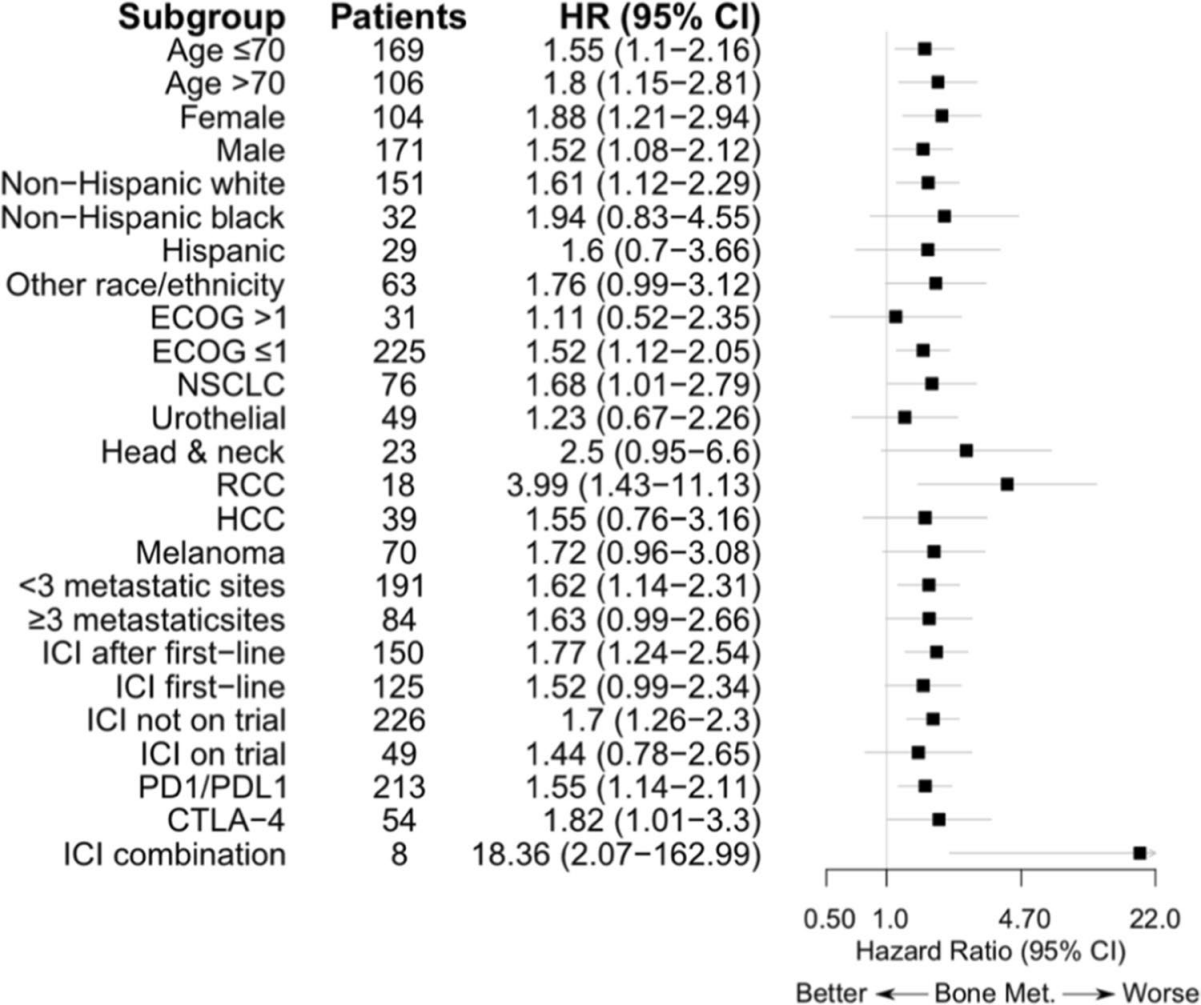
Association of Bone Metastases with PFS across Clinical Subgroups

Given the observation that the presence of bone metastasis was a potential predictor of worse PFS, ORR, and DCR in our regression models, we conducted subgroup analyses to examine the association of bone metastases with the PFS across various demographic and clinical subgroups (Fig. 1, Supplemental Figure 2). Bone metastases were consistently associated with worse PFS across subgroups defined by age, sex, and primary malignancy (Fig. 1, Supplemental Figure 2). There were no statistically significant interactions to indicate the presence of effect modification within any subgroup.

## Discussion

Prior studies have explored associations between clinical characteristics and ICI response, although results are widely variable and often inconclusive. For example, as many studies report a favorable correlation between older age and ICI response as those suggesting the opposite [5, 9–12, 16]. Meta-analyses such as those by Conforti et al. and Wu et al. found significantly higher PFS and/or OS benefit in males treated with ICI versus control when compared to females treated with ICI versus control, possibly attributed to sex dimorphism in immunity and cancer biology [8, 17]. However, the sex-related advantage seems to vary according to tumor type and class of ICI, among other factors [18, 19]. For example, higher portion of male are smokers when compared to female, and smoking has been associated with predictive biomarkers such as TMB [20, 21]. Alternative host characteristics explored in the literature include obesity, performance status, co-morbidities, and more [6, 7, 12, 22–24].

In our analysis of host characteristics, age, race, and sex were not associated with differences in ICI efficacy or OS; nor were obesity, smoking, or comorbidities such as kidney disease when evaluated in multivariable models. However, performance status was a significant clinical parameter: ECOG ≤ 2 correlated with both improved ICI response (PFS, ORR, and DCR) and OS. This is in line with a recent study by Dall’Olio and colleagues, where a meta-analysis of 19 retrospective and prospective studies correlated performance status ≥ 2 with worse ICI response (ORR, PFS) and OS in 3,600 NSCLC patients [6]. In contrast, the meta-analysis of 18 clinical trials incorporating patients across a variety of tumor types showed no difference in OS among ICI-treated patients with ECOG 0 versus ECOG of 1–2 [7]. The exclusion of poor performance status patients from clinical trials may partly explain the lack of correlation seen in the latter meta-analysis, where only 11 out of 11,354 patients evaluated had an ECOG ≥ 2 [7]. This essentially makes the meta-analysis a comparison of ECOG 0 versus 1, which likely has insufficient clinical fitness differences to alter ICI treatment outcomes. Our study supports the inferior effects of ICIs in solid tumor patients with ECOG ≥ 2 and highlights the importance of real-world data. More importantly, when both ICI and chemotherapy are appropriate therapeutic options, clinical practice often favors ICI in frail patients, driven by the favorable toxicity profile of ICIs and higher toxicity/inferior benefits of chemotherapy in patients with poor performance status [12, 25–27]. However, our study suggests that ICI efficacy is relatively compromised in patients with poor performance status, highlighting the importance of patient-centered discussions of risk, benefits, and preferences when selecting treatment for these vulnerable patients.

Interestingly, family history of cancer was also associated with improved ORR and DCR to ICI therapy, hinting at underlying genetic components to immunogenicity. In the multicenter retrospective study of 211 advanced cancer patients, Cortellini and colleagues also found statistically significant correlation between family history of cancer and ICI response with improved ORR (p = 0.0024), DCR (p = 0.0161), median time to treatment failure (p = 0.0203), and median OS (p-0.0221) [28]. Expanded analyses of 811 advanced cancer patients upheld family history of cancer as an independent predictor of PFS and OS in multivariable analyses [29]. Our data supports their assessment that family history of cancer may be a surrogate for known (and yet to be identified) syndromes of inherited cancer susceptibility. Such syndromes (e.g. Lynch syndrome) are often characterized by deficient mismatch repair and characteristic high levels of microsatellite instability, leading to higher TMB and potentially more immunogenic tumors with higher response to ICI therapy [30–33]. Further studies correlating family history of cancer, germline genetic data, and ICI response are worth exploring. In the clinical setting, germline testing is certainly warranted in advanced, solid tumor patients with family history of cancer, and should be discussed in accordance with guidelines in patients without family history of cancer.

In a review of literature, several studies across a variety of tumor types correlated bone metastasis with worse ICI response and/or OS (Table 3) [34–41]. In our analysis of tumor-specific characteristics, bone metastasis was associated with worse ICI response (PFS, ORR, and DCR). Furthermore, subgroup analysis consistently associated bone metastasis with worse PFS regardless of age, gender, race, performance status, tumor type, number of metastatic sites, ICI as first or subsequent lines, type of ICI, or combination versus single agent ICI (Fig. 1). Particularly, the negative impact of bone metastasis regardless of the primary tumor type suggests a unique immunologic niche in the bone microenvironment. In their analysis of pre- and post-ipilimumab bone marrow tissues from patients with metastatic castration-resistant prostate cancer (mCRPC), Jiao and colleagues noted the absence of Th1 lineage expansion in bone metastasis versus their presence in soft tissue metastases [42]. To further explore, Jiao and colleagues compared mice models with bone mCRPC versus subcutaneous mCRPC and found anti-CTLA-4/anti-PD-1 antibodies had minimal effects on tumor volume and OS in mice bearing the bone CRPC lesions but significant regression and improved OS in the mice with subcutaneous CRPC [42]. Deeper analysis of the bone-specific tumor microenvironment and larger studies evaluating the consequence of bone metastasis on ICI response may guide its future role as a predictive and/or prognostic biomarker. Furthermore, combination therapy (ie, ICI with chemotherapy, targeted agents, with or without bone-targeted agents such as denosumab) are increasingly being explored across a variety of tumor types. A focus on the effect of such combinations on bone metastasis should be considered.

**Table 3:** literature Review on the Association of Bone Metastasis with Response to Immune Checkpoint Inhibitors

Study	Patients	Met site	N	ORR	PFS	OS
MSH	297 NSCLC, Melanoma, HNSC, HCC, UC, RCC	Bone	102	Worse	Worse	NS
Tamiya [31]	NSCLC	Bone	66	NA	NS	NA
Garde-Noguera [32]	175 NSCLC	Bone	67	NA	NS	NS
Bilen [33]	90 Melanoma, GI	Bone	24	NS (DCR, Uni only)	NS (Uni only)	NS (Uni only)
Landi [34]	1588 NSCLC	Bone	626	Worse	Worse	Worse
Cortellini [35]	1026 PDL1-hi NSCLC	Bone	272	Worse	Worse	Worse
Botticelli [36]	291 NSCLC, Melanoma, RCC	Bone	75	NA	Worse (Uni only)	Worse (Uni only)
Kawachi [37]	213 PDL1-hi NSCLC	Bone	59	NA	NS (Uni only)	NA
Gomez de Liano Lista [38]	270 UC	Bone		NA	NA	Worse

*GI* gastrointestinal; *HCC* hepatocellular carcinoma; *HNSC* head and neck squamous cell carcinoma; *MSH* Mount Sinai Hospital (current study); *NA* Not available; *NS* Not significant; *NSCLC* non-small cell lung cancer; *RCC* PDL1-hi programmed death-ligand 1 high; renal cell carcinoma; *UC* urothelial carcinoma; *Uni only* only univariate result reported

Limitations to our study include its single academic center and retrospective nature which is inherently subject to selection bias. Certain clinical factors of interest, including patients receiving single dose of ICI, blood-based biomarkers (such as neutrophil–lymphocyte ratio), comorbidities (such as cardiac, pulmonary, or liver disease), were not included due to limitations associated with the data availability and/or sample size (i.e., insufficient sample and/or abnormal values leading to insufficient statistical strength). Additional limitations include heterogeneity of baseline characteristics and therapeutic settings across tumor types. Specifically, difference in baseline characteristics such as age, sex and smoking status have the potential to influence outcomes, although we attempted to mitigate these differences through multivariable analysis. Furthermore, inclusion of multiple tumor types, small sample size in certain cancers (i.e., HNSC and RCC, as well as small proportion of male patients with lung cancer), and various lines of ICI therapy may limit our ability to draw conclusive PFS and OS correlations. Similarly, although clinical characteristics of performance status, family history of cancer, and bone metastasis may be prognostic, they cannot be deemed predictive within the scope of this retrospective analysis. Subgroup analyses are largely hypothesis generating due to the risk of both false positives as a result of multiple comparisons as well as false negatives arising from reduced statistical power, we propose that the results of subgroup analyses herein have to be confirmed by further research [43]. Lastly, genomic data, including hereditary and familial syndrome testing, is inadequately evaluated in the current study and should be considered in future studies given the association of family history of cancer with ICI outcomes.

## Conclusion

In this study exploring the associations between clinical characteristics and ICI response, good performance status (ECOG ≤ 2) and family history of cancer correlated with improved ICI outcomes while bone metastasis correlated with worse ICI outcomes. Our study adds real-world data exploring clinical associations, which remains relatively lacking in published literature. Underlying mechanisms for these observations, such as genetic variants and bone metastasis-specific tumor microenvironment, may be worth exploring in larger studies incorporating translational endpoints. Validation of these clinical factors from larger real-world data sets may help inform future treatment selection when considering ICI therapy.

## Supplementary Information

**Additional file 1.**

**Additional file 2.**

## Data Availability

The datasets generated during and/or analysed during the current study are available from the corresponding author on reasonable request.

## References

[1] Gong J, Chehrazi-Raffle A, Reddi S, Salgia R (2018). Development of PD-1 and PD-L1 inhibitors as a form of cancer immunotherapy: a comprehensive review of registration trials and future considerations. J Immunother Cancer.

[2] Hodi FS, O'Day SJ, McDermott DF, Weber RW, Sosman JA, Haanen JB (2010). Improved survival with ipilimumab in patients with metastatic melanoma. N Engl J Med.

[3] Bai R, Lv Z, Xu D, Cui J (2020). Predictive biomarkers for cancer immunotherapy with immune checkpoint inhibitors. Biomark Res.

[4] Litchfield K, Reading JL, Puttick C, Thakkar K, Abbosh C, Bentham R (2021). Meta-analysis of tumor- and T cell-intrinsic mechanisms of sensitization to checkpoint inhibition. Cell.

[5] Yan X, Tian X, Wu Z, Han W (2020). Impact of age on the efficacy of immune checkpoint inhibitor-based combination therapy for non-small-cell lung cancer: a systematic review and meta-analysis. Front Oncol.

[6] Dall'Olio FG, Maggio I, Massucci M, Mollica V, Fragomeno B, Ardizzoni A (2020). ECOG performance status >/=2 as a prognostic factor in patients with advanced non small cell lung cancer treated with immune checkpoint inhibitors-a systematic review and meta-analysis of real world data. Lung Cancer.

[7] Bersanelli M, Brighenti M, Buti S, Barni S, Petrelli F (2018). Patient performance status and cancer immunotherapy efficacy: a meta-analysis. Med Oncol.

[8] Conforti F, Pala L, Bagnardi V, De Pas T, Martinetti M, Viale G (2018). Cancer immunotherapy efficacy and patients' sex: a systematic review and meta-analysis. Lancet Oncol.

[9] Fulop T, Larbi A, Kotb R, de Angelis F, Pawelec G (2011). Aging, immunity, and cancer. Discov Med.

[10] Kugel CH, Douglass SM, Webster MR, Kaur A, Liu Q, Yin X (2018). Age correlates with response to anti-PD1, reflecting age-related differences in intratumoral effector and regulatory T-Cell populations. Clin Cancer Res.

[11] Nishijima TF, Muss HB, Shachar SS, Moschos SJ (2016). Comparison of efficacy of immune checkpoint inhibitors (ICIs) between younger and older patients: A systematic review and meta-analysis. Cancer Treat Rev.

[12] Felip E, Ardizzoni A, Ciuleanu T, Cobo M, Laktionov K, Szilasi M (2020). CheckMate 171: a phase 2 trial of nivolumab in patients with previously treated advanced squamous non-small cell lung cancer, including ECOG PS 2 and elderly populations. Eur J Cancer.

[13] Eisenhauer EA, Therasse P, Bogaerts J, Schwartz LH, Sargent D, Ford R (2009). New response evaluation criteria in solid tumours: revised RECIST guideline (version 1.1). Eur J Cancer.

[14] Harris PA, Taylor R, Minor BL, Elliott V, Fernandez M, O'Neal L (2019). The REDCap consortium: building an international community of software platform partners. J Biomed Inform.

[15] Harris PA, Taylor R, Thielke R, Payne J, Gonzalez N, Conde JG (2009). Research electronic data capture (REDCap)–a metadata-driven methodology and workflow process for providing translational research informatics support. J Biomed Inform.

[16] Daste A, Domblides C, Gross-Goupil M, Chakiba C, Quivy A, Cochin V (2017). Immune checkpoint inhibitors and elderly people: a review. Eur J Cancer.

[17] Wu Y, Ju Q, Jia K, Yu J, Shi H, Wu H (2018). Correlation between sex and efficacy of immune checkpoint inhibitors (PD-1 and CTLA-4 inhibitors). Int J Cancer.

[18] Ye Y, Jing Y, Li L, Mills GB, Diao L, Liu H (2020). Sex-associated molecular differences for cancer immunotherapy. Nat Commun.

[19] Wang S, Cowley LA, Liu XS (2019). Sex differences in cancer immunotherapy efficacy, biomarkers, and therapeutic strategy. Molecules.

[20] Davis AA, Chae YK, Agte S, Pan A, Mohindra NA, Villaflor VM (2017). Association of tumor mutational burden with smoking and mutation status in non-small cell lung cancer (NSCLC). J Clin Oncol.

[21] Lee KWC, Lord SJ, Kasherman L, Marschner I, Stockler M, Gralla R (2020). The impact of smoking on the effectiveness of immune checkpoint inhibitors—a systematic review and meta-analysis. Acta Oncol.

[22] Dudnik E, Moskovitz M, Daher S, Shamai S, Hanovich E, Grubstein A (2018). Effectiveness and safety of nivolumab in advanced non-small cell lung cancer: the real-life data. Lung Cancer.

[23] Murphy WJ, Longo DL (2019). The surprisingly positive association between obesity and cancer immunotherapy efficacy. JAMA.

[24] Wang Z, Aguilar EG, Luna JI, Dunai C, Khuat LT, Le CT (2019). Paradoxical effects of obesity on T cell function during tumor progression and PD-1 checkpoint blockade. Nat Med.

[25] Sweeney CJ, Zhu J, Sandler AB, Schiller J, Belani CP, Langer C (2001). Outcome of patients with a performance status of 2 in Eastern Cooperative Oncology Group Study E1594: a Phase II trial in patients with metastatic nonsmall cell lung carcinoma. Cancer.

[26] Di Maio M, Lama N, Morabito A, Smit EF, Georgoulias V, Takeda K (2010). Clinical assessment of patients with advanced non-small-cell lung cancer eligible for second-line chemotherapy: a prognostic score from individual data of nine randomised trials. Eur J Cancer.

[27] Caires-Lima R, Cayres K, Protasio B, Caires I, Andrade J, Rocha L (2018). Palliative chemotherapy outcomes in patients with ECOG-PS higher than 1. Ecancermedicalscience.

[28] Cortellini A, Bersanelli M, Buti S, Gambale E, Atzori F, Zoratto F (2018). Family history of cancer as surrogate predictor for immunotherapy with anti-PD1/PD-L1 agents: preliminary report of the FAMI-L1 study. Immunotherapy.

[29] Cortellini A, Buti S, Bersanelli M, Giusti R, Perrone F, Di Marino P (2020). Evaluating the role of FAMIly history of cancer and diagnosis of multiple neoplasms in cancer patients receiving PD-1/PD-L1 checkpoint inhibitors: the multicenter FAMI-L1 study. Oncoimmunology.

[30] Latham A, Srinivasan P, Kemel Y, Shia J, Bandlamudi C, Mandelker D (2019). Microsatellite instability is associated with the presence of lynch syndrome pan-cancer. J Clin Oncol.

[31] Chalmers ZR, Connelly CF, Fabrizio D, Gay L, Ali SM, Ennis R (2017). Analysis of 100,000 human cancer genomes reveals the landscape of tumor mutational burden. Genome Med.

[32] Goodman AM, Kato S, Bazhenova L, Patel SP, Frampton GM, Miller V (2017). Tumor mutational burden as an independent predictor of response to immunotherapy in diverse cancers. Mol Cancer Ther.

[33] Kim JY, Kronbichler A, Eisenhut M, Hong SH, van der Vliet HJ, Kang J (2019). Tumor mutational burden and efficacy of immune checkpoint inhibitors: a systematic review and meta-analysis. Cancers (Basel)..

[34] Tamiya M, Tamiya A, Inoue T, Kimura M, Kunimasa K, Nakahama K (2018). Metastatic site as a predictor of nivolumab efficacy in patients with advanced non-small cell lung cancer: a retrospective multicenter trial. PLoS ONE.

[35] Garde-Noguera J, Martin-Martorell P, De Julian M, Perez-Altozano J, Salvador-Coloma C, Garcia-Sanchez J (2018). Predictive and prognostic clinical and pathological factors of nivolumab efficacy in non-small-cell lung cancer patients. Clin Transl Oncol.

[36] Bilen MA, Shabto JM, Martini DJ, Liu Y, Lewis C, Collins H (2019). Sites of metastasis and association with clinical outcome in advanced stage cancer patients treated with immunotherapy. BMC Cancer.

[37] Landi L, D'Inca F, Gelibter A, Chiari R, Grossi F, Delmonte A (2019). Bone metastases and immunotherapy in patients with advanced non-small-cell lung cancer. J Immunother Cancer.

[38] Cortellini A, Tiseo M, Banna GL, Cappuzzo F, Aerts J, Barbieri F (2020). Clinicopathologic correlates of first-line pembrolizumab effectiveness in patients with advanced NSCLC and a PD-L1 expression of ≥ 50. Cancer Immunol Immunother.

[39] Botticelli A, Cirillo A, Scagnoli S, Cerbelli B, Strigari L, Cortellini A (2020). The agnostic role of site of metastasis in predicting outcomes in cancer patients treated with immunotherapy. Vaccines (Basel)..

[40] Kawachi H, Tamiya M, Tamiya A, Ishii S, Hirano K, Matsumoto H (2020). Association between metastatic sites and first-line pembrolizumab treatment outcome for advanced non-small cell lung cancer with high PD-L1 expression: a retrospective multicenter cohort study. Invest New Drugs.

[41] de Liano G, Lista A, van Dijk N, de Velasco Oria de Rueda G, Necchi A, Lavaud P, Morales-Barrera R (2020). Clinical outcome after progressing to frontline and second-line Anti-PD-1/PD-L1 in advanced urothelial cancer. Eur Urol.

[42] Jiao S, Subudhi SK, Aparicio A, Ge Z, Guan B, Miura Y (2019). Differences in tumor microenvironment dictate T helper lineage polarization and response to immune checkpoint therapy. Cell.

[43] Burke JF, Sussman JB, Kent DM, Hayward RA (2015). Three simple rules to ensure reasonably credible subgroup analyses. BMJ.

