# Supplementary material for: Clinical factors associated with outcome in solid tumor patients treated with immune-checkpoint inhibitors: a single institution retrospective analysis
Source: Discov Oncol. 2022 Aug 12;13:73. doi: 10.1007/s12672-022-00538-6 (PMC9374856; doi:10.1007/s12672-022-00538-6)
Supplement: Supplementary file 1 — Additional file 1. [file 12672_2022_538_MOESM1_ESM.docx]

**Additional file 1: Figure S1. Survival Outcomes by Primary Malignancy**


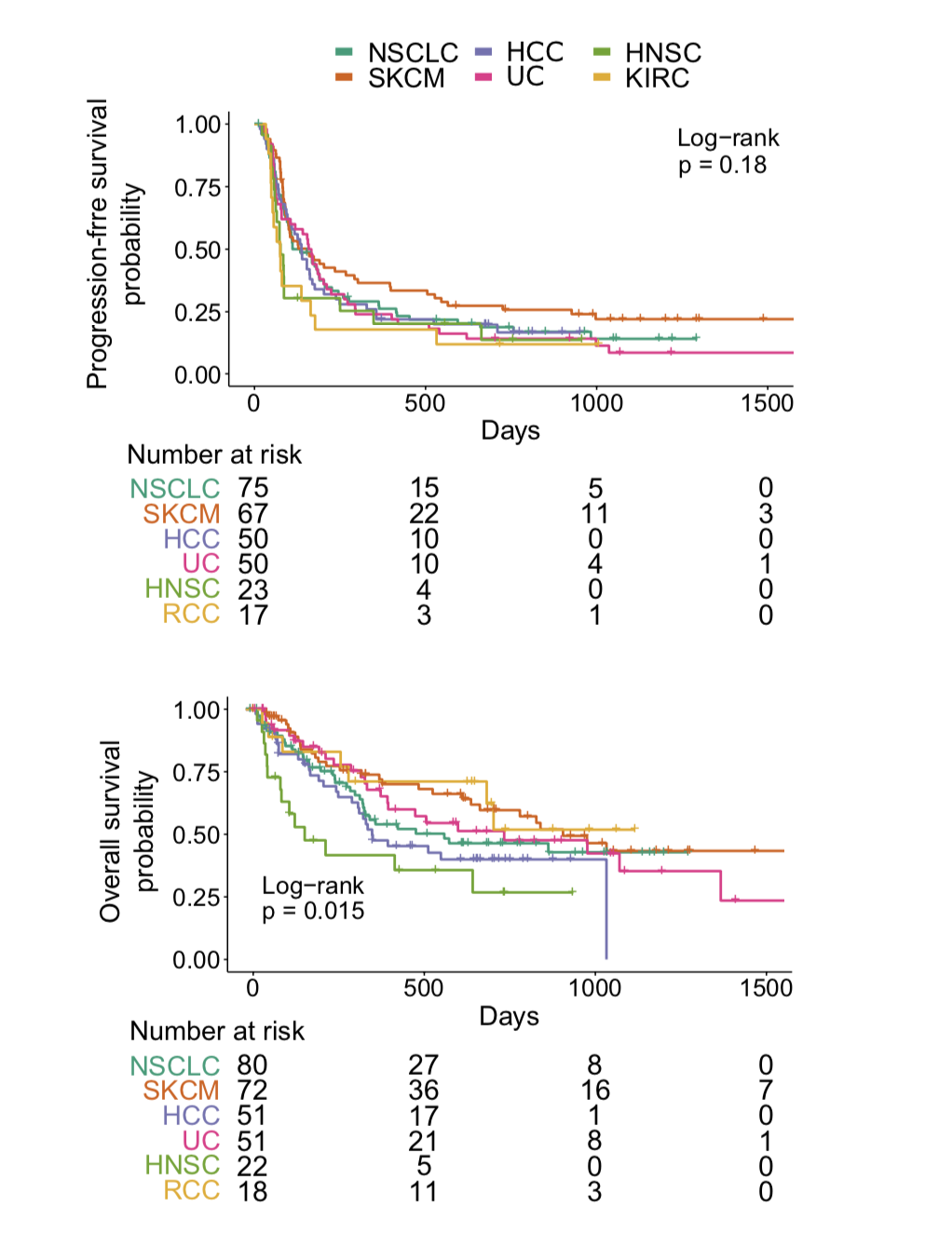


HCC: Hepatocellular carcinoma; HNSC: Head and neck squamous cancer; NSCLC: Non-small cell lung cancer; RCC: Renal cell carcinoma; UC Urothelial carcinoma

**Additional file 1: Figure S2. Bone Metastasis-Associated Progression Free Survival and Overall Survival Outcomes by Primary Malignancy**

1.
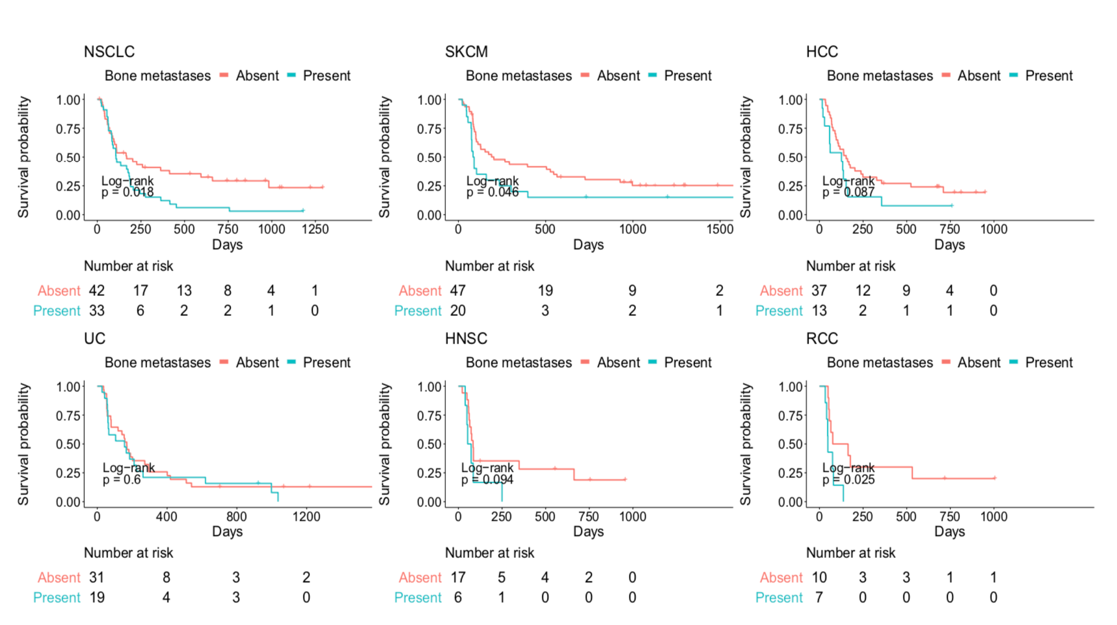
Progression Free Survival
2.
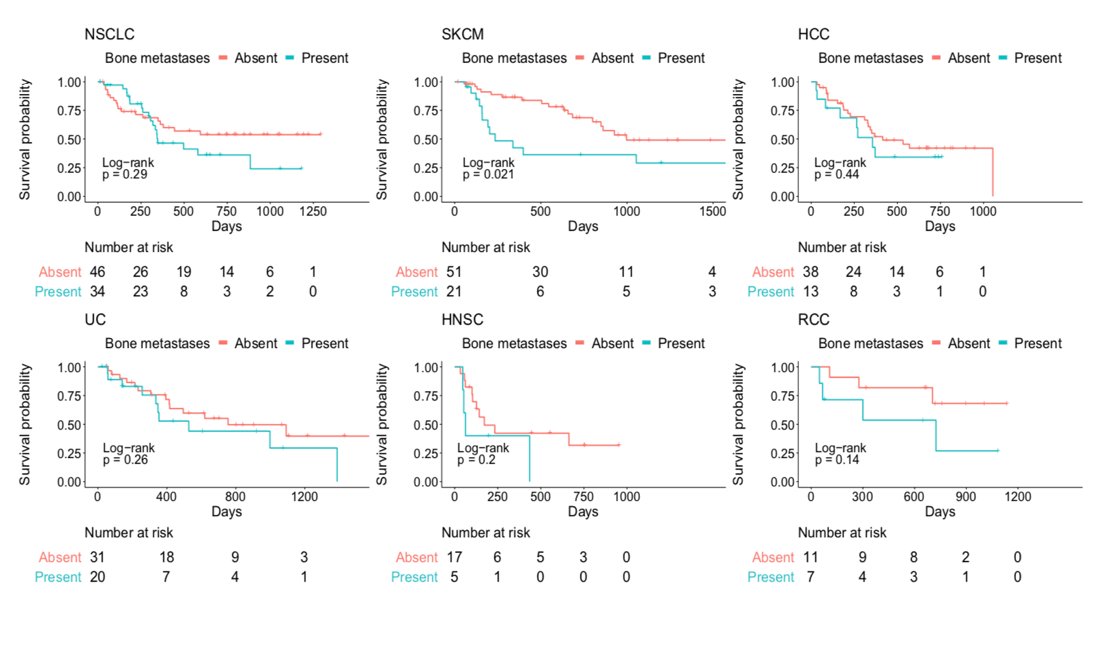
Overall Survival

HCC: Hepatocellular carcinoma; HNSC: Head and neck squamous cancer; NSCLC: Non-small cell lung cancer; RCC: Renal cell carcinoma; SKCM: melanoma; UC Urothelial carcinoma
