# Supplementary material for: Clinical factors associated with outcome in solid tumor patients treated with immune-checkpoint inhibitors: a single institution retrospective analysis
Source: Discov Oncol. 2022 Aug 12;13:73. doi: 10.1007/s12672-022-00538-6 (PMC9374856; doi:10.1007/s12672-022-00538-6)
Supplement: Supplementary file 2 — Additional file 2. [file 12672_2022_538_MOESM2_ESM.docx]

Additional file 2: Table S1: Median Survival Values by Cancer Type

| Cancer | Outcome | Median survival (days) |
| --- | --- | --- |
| NSCLC | PFS | 122.5 |
| Melanoma | PFS | 153 |
| HCC | PFS | 137.5 |
| UC | PFS | 162 |
| HNSC | PFS | 76 |
| RCC | PFS | 75 |
| All cancers | PFS | 128 |
| NSCLC | OS | 580 |
| Melanoma | OS | 927 |
| HCC | OS | 369 |
| UC | OS | 755 |
| HNSC | OS | 172 |
| RCC | OS | Not reached |
| All cancers | OS | 663 |

HCC: Hepatocellular carcinoma; HNSC: Head and neck squamous cancer; NSCLC: Non-small cell lung cancer; OS: overall survival; PFS: progression free survival; RCC: Renal cell carcinoma; UC Urothelial carcinoma.

Additional file 2: Table S2: Unadjusted and Adjusted Logistic Regression Models Predicting Overall Response

| **Variable** | **Unadjusted OR**  **(95% CI)** | **Age, sex, tumor-adjusted OR**  **(95% CI)** | **Multivariable model**  **OR**† **(95% CI)** |
| --- | --- | --- | --- |
| Age (yrs) | 1.03 (1-1.05)* | 1.02 (1-1.05) | 1.02 (1-1) |
| Male | 0.73 (0.42-1.25) | 0.8 (0.45-1.44) | 0.88 (0.47-1.7) |
| Non-Hispanic black | 1.49 (0.68-3.29) | 1.82 (0.76-4.33) | - |
| Hispanic | 0.47 (0.18-1.24) | 0.53 (0.19-1.47) | - |
| Other | 0.54 (0.27-1.08) | 0.71 (0.34-1.51) | - |
| ECOG 0-1 | 6.17 (1.41-26.81)* | 8.33 (1.81-38.07)* | 7.39 (1.6-35)* |
| Body mass index | 1.02 (0.97-1.06) | 1.02 (0.97-1.07) | - |
| Family history of cancer | 2.03 (1.2-3.48)* | 1.95 (1.11-3.45)* | 1.93 (1-3.6)* |
| Current or former smoker | 1.57 (0.87-2.82) | 1.52 (0.8-2.88) | - |
| Est. GFR <60 | 1 (0.55-1.82) | 0.99 (0.49-2) | - |
| Locally advanced | 1.3 (0.45-3.69) | 2.2 (0.68-7.06) | - |
| 3 or more metastatic sites | 0.76 (0.42-1.38) | 0.59 (0.32-1.12) | - |
| Lung metastases | 0.65 (0.36-1.16) | 0.67 (0.36-1.25) | - |
| Liver metastases | 0.8 (0.4-1.58) | 0.77 (0.38-1.56) | - |
| Lymph node metastases | 1.3 (0.74-2.25) | 1.09 (0.61-1.97) | - |
| Bone metastases | 0.42 (0.23-0.76)* | 0.39 (0.21-0.73)* | 0.41 (0.21-0.81)* |
| CNS metastases | 1.21 (0.55-2.66) | 1.02 (0.44-2.4) | - |
| Other metastases | 1.02 (0.56-1.86) | 0.9 (0.47-1.69) | - |
| ICI given first line | 1.49 (0.88-2.52) | 1.34 (0.73-2.47) | - |
| ICI given on trial | 1.57 (0.82-3.02) | 1.72 (0.7-4.17) | - |
| CTLA-4 | 1.17 (0.61-2.27) | 0.79 (0.3-2.1) | - |
| ICI combination | 1.32 (0.31-5.72) | 0.99 (0.18-5.28) | - |

CI: confidence interval; CNS: central nervous system; CTLA-4: cytotoxic T-lymphocyte-associated protein 4; ECOG: Eastern Cooperative Oncology Group; est: estimated; GFR: glomerular filtration rate; ICI: immune checkpoint inhibitor; OR: odds ratio; PD-1: programmed cell death protein 1; PD-L1: programmed death-ligand 1; yrs: years. *:p<0.05. †: Multivariable models also adjusted for tumor histology.

Additional file 2: Table S3: Unadjusted and Adjusted Logistic Regression Models Predicting Disease Control

| **Variable** | **Unadjusted OR**  **(95% CI)** | **Age, sex, tumor-adjusted OR**  **(95% CI)** | **Multivariable model**  **OR**† **(95% CI)** |
| --- | --- | --- | --- |
| Age (yrs) | 1.02 (1-1.04)* | 1.02 (1-1.04) | 1.02 (1-1) |
| Male | 0.69 (0.41-1.15) | 0.68 (0.39-1.17) | 0.66 (0.36-1.2) |
| Non-Hispanic black | 1.13 (0.51-2.47) | 1.57 (0.66-3.7) | - |
| Hispanic | 0.35 (0.15-0.85)* | 0.41 (0.16-1.03) | - |
| Other | 0.84 (0.46-1.54) | 1.08 (0.55-2.14) | - |
| ECOG 0-1 | 4.14 (1.5-11.45)* | 4.9 (1.69-14.25)* | 3.67 (1.3-11)* |
| Body mass index | 1.04 (1-1.09) | 1.03 (0.99-1.09) | - |
| Family history of cancer | 2.12 (1.29-3.51)* | 2.25 (1.31-3.84)* | 2.14 (1.2-3.8)* |
| Current or former smoker | 1.51 (0.89-2.57) | 1.75 (0.97-3.15) | - |
| Est. GFR <60 | 1.19 (0.67-2.07) | 0.88 (0.45-1.71) | - |
| Locally advanced | 2.48 (0.83-7.33) | 3.6 (1.1-11.64)* | 2.46 (0.74-8.3) |
| 3 or more metastatic sites | 0.84 (0.49-1.44) | 0.74 (0.41-1.33) | - |
| Lung metastases | 0.67 (0.39-1.15) | 0.65 (0.36-1.17) | - |
| Liver metastases | 0.73 (0.39-1.38) | 0.68 (0.35-1.33) | - |
| Lymph node metastases | 1.15 (0.69-1.9) | 1.11 (0.64-1.92) | - |
| Bone metastases | 0.44 (0.26-0.75)* | 0.42 (0.24-0.73)* | 0.45 (0.24-0.83)* |
| CNS metastases | 0.87 (0.41-1.85) | 0.98 (0.43-2.23) | - |
| Other metastases | 1.55 (0.88-2.73) | 1.35 (0.74-2.45) | - |
| ICI given first line | 1.54 (0.94-2.53) | 1.43 (0.8-2.54) | - |
| ICI given on trial | 1.55 (0.82-2.95) | 1.12 (0.47-2.66) | - |
| CTLA-4 | 1.28 (0.69-2.41) | 1.14 (0.43-3) | - |
| ICI combination | 1.9 (0.44-8.16) | 1.93 (0.35-10.79) | - |

CI: confidence interval; CNS: central nervous system; CTLA-4: cytotoxic T-lymphocyte-associated protein 4; ECOG: Eastern Cooperative Oncology Group; est: estimated; GFR: glomerular filtration rate; ICI: immune checkpoint inhibitor; OR: odds ratio; PD-1: programmed cell death protein 1; PD-L1: programmed death-ligand 1; yrs: years. *:p<0.05. †: Multivariable models also adjusted for tumor histology.

*:p<0.05. †: Multivariable models also adjusted for tumor histology.
